# Mid-trimester resolution of marked dextroposition of the fetal heart preceding regression of extensive left fetal lung lesion consisting of hybrid congenital pulmonary airway malformation (CPAM) and bronchopulmonary sequestration (BPS)

**DOI:** 10.1016/j.radcr.2024.11.078

**Published:** 2025-01-07

**Authors:** David M. Sherer, Ida Dhanuka, Kayla Schacher, Hubert Rodriguez-Tejada, Aleksandra Zigalo, Mila Kheyman, Harry Zinn, Fancisca T. Velcek, Mudar Dalloul

**Affiliations:** a Division of Maternal Fetal Medicine, Department of Obstetrics and Gynecology, State University of New York (SUNY), Downstate Health Sciences University, Brooklyn, NY, USA; b Department of Radiology, State University of New York (SUNY), Downstate Health Sciences University, Brooklyn, NY, USA; c Division of Pediatric Surgery, Department of Surgery, State University of New York (SUNY), Downstate Health Sciences University, Brooklyn, NY, USA

**Keywords:** Prenatal ultrasound, Congenital pulmonary airway malformation (CPAM), Bronchopulmonary sequestration (BPS), Hybrid lung lesion, Dextroposition of the fetal heart, Cardiomediastinal shift angle

## Abstract

Extensive congenital pulmonary airway malformation (CPAM) of the left fetal lung and associated marked dextroposition of the fetal heart were noted at 21 weeks’ gestation. The right fetal lung appeared compressed with the cardiomediastinal shift angle measuring approximately 20 degrees. Potential subsequent right pulmonary hypoplasia was considered. At 26 weeks’ gestation, despite the continued presence of extensive CPAM of the left fetal lung, spontaneous resolution of the dextroposition of the fetal heart was noted. Subsequent repeat ultrasound assessments confirmed gradual continued in-utero regression of the left lung lesion. The patient spontaneously delivered a vigorous infant neonate at 39 and 4/7 weeks’ gestation. Birth weight was 3175 grams, and following brief CPAP management for mild transient tachypnea of the newborn, and negative chest X-ray, the infant was discharged in good health on Day 2 of life. Neonatal CT angiography demonstrated CPAM of the left lower lobe. In addition, a feeding vessel was seen emanating directly from the thoracic aorta, indicating an intralobar brochopulmonary sequestration (BPS) component of the left lung lesion, consistent with a hybrid lung lesion (CPAM and intralobar BPS). At 2 months of life the infant underwent uneventful resection of the left lower lobe with pathology confirmation of the hybrid lung lesion. This case demonstrates that relatively rapid regression of severe cardiomediastinal shift associated with extensive hybrid lung lesions may occur. Our case indicates that spontaneous regression of marked cardiomediastinal shift appears to be a reassuring prognostic sign despite the continued presence of this lesion.

## Introduction

Congenital pulmonary airway malformation (CPAM) is a well-described fetal structural anomaly [[1], [2], [3]]. This condition is the most common congenital lung lesion with a reported incidence of 0.94 in 10,000 live births accounting for approximately 95% of all congenital lung lesions and in most cases is limited to 1 lobe [[1], [2], [3], [4], [5]]. Differential diagnosis includes: bronchopulmonary sequestration (BPS), congenital lobar over-inflation, emphysema, bronchogenic cyst and bronchial atresia [2]. Clinical prognosis of CPAM is highly variable, and ranges from apparent in-utero resolution (in approximately 30%-50% of cases), to the continued severe mass effect, which may result in hydrops fetalis and stillbirth [[1], [2], [3], [4]]. We report a case of extensive hybrid lesion (CPAM and BPS) of the left fetal lung associated with considerable right-sided displacement (dextroposition) of the fetal heart, reflecting the mass effect of the extensive left-sided hybrid fetal lung lesion. The cardiomediastinal shift angle (CMSA), an objective sonographic measurement of cardiomediastinal shift described originally in 2019 by Shulman et al in association with this lesion, measured approximately 20 degrees [3]. Spontaneous in-utero resolution of the severe cardiomediastinal shift 5 weeks later preceded subsequent gradual continued regression of the extensive fetal lung lesion. A vigorous asymptomatic neonate was born at term. Neonatal CT angiography demonstrated a hybrid intrapulmonary bronchopulmonary sequestration and congenital pulmonary malformation of left lower lobe. The infant underwent resection of the left lower lobe. Pathology confirmed the hybrid lung lesion.

## Case report

**Fig. 1:**
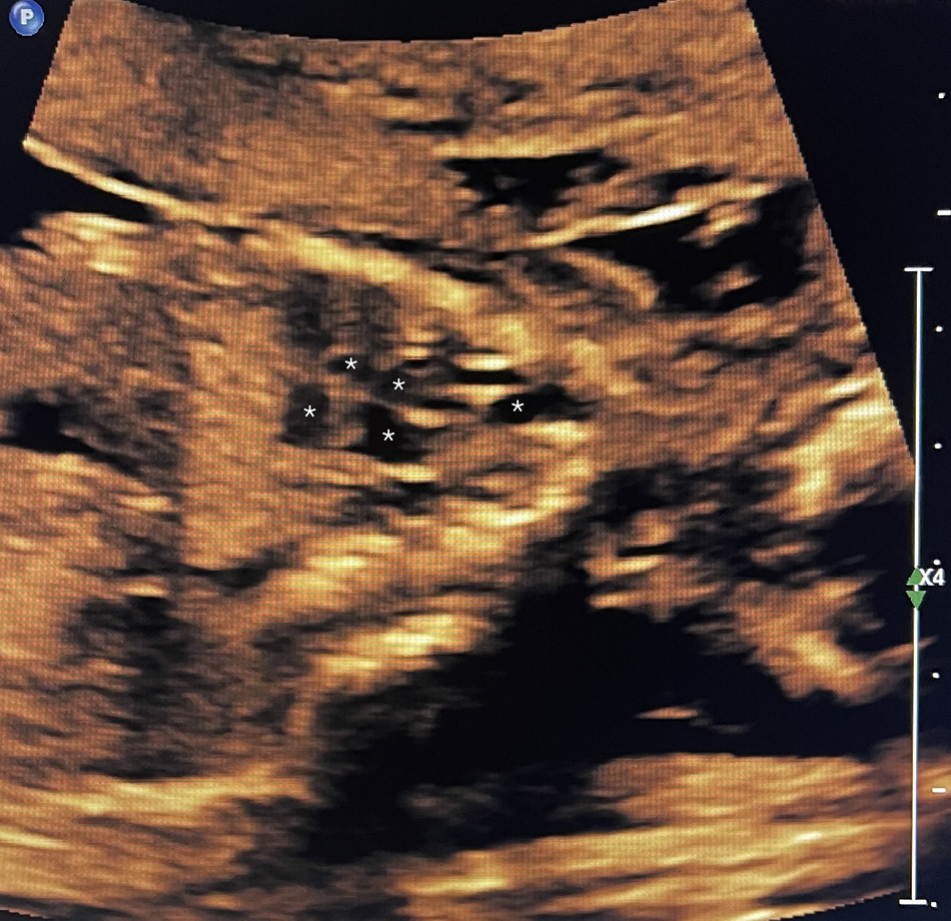
Sagittal sonographic image of the left fetal thorax at 21 weeks’ gestation. Note the extensive presence of cystic structures of varying sizes (mixed microcystic and macrocystic lesions) throughout the left fetal lung.

**Fig. 2:**
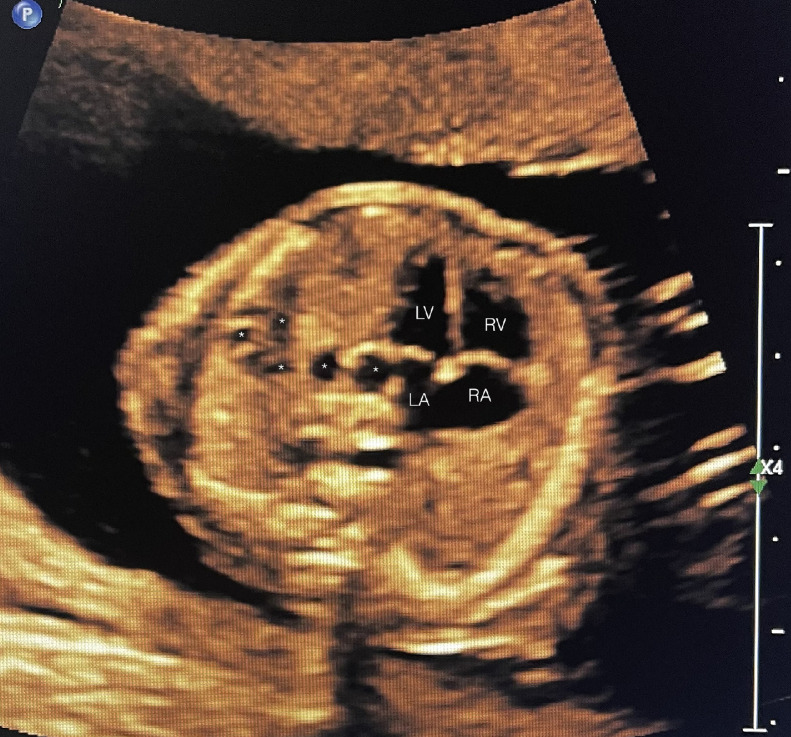
Axial sonographic image of the fetal thorax at the level of the 4-chamber heart, depicted at 21 weeks’ gestation. Note the marked displacement of the fetal heart to the right - dextro-position of the fetal heart. The fetal heart essentially occupies the right hemithorax. LV, LA, RV, RA, designate the respective cardiac chambers. Note also the compressed and barely visible right fetal lung.

**Fig. 3:**
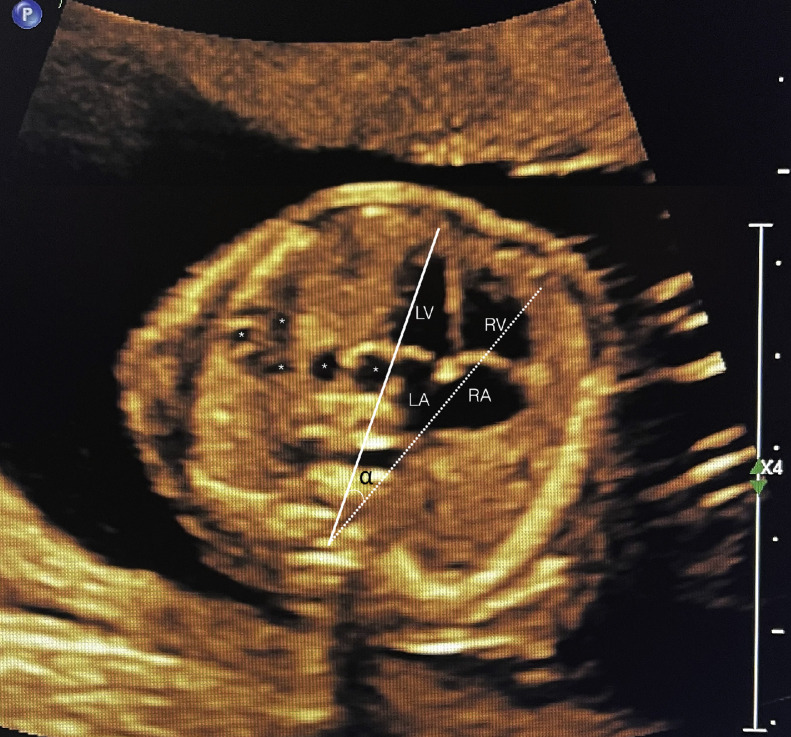
The identical axial sonographic image of the fetal thorax at the level of the four-chamber heart depicted at 21 weeks’ gestation, as in Fig. 2. This figure contains lines defining the degree of displacement of the fetal heart to the right (dextroposition). The midline (solid line) in the normal-positioned heart would have traversed the right cardiac chambers (RA, RV). However, in this dextropositioned fetal heart this line has been considerably displaced to the right, and is represented by the dotted line. The angle between the two lines solid and dotted (marked with a) represents the cardiomediastinal shift angle (CMSA).

**Fig. 4:**
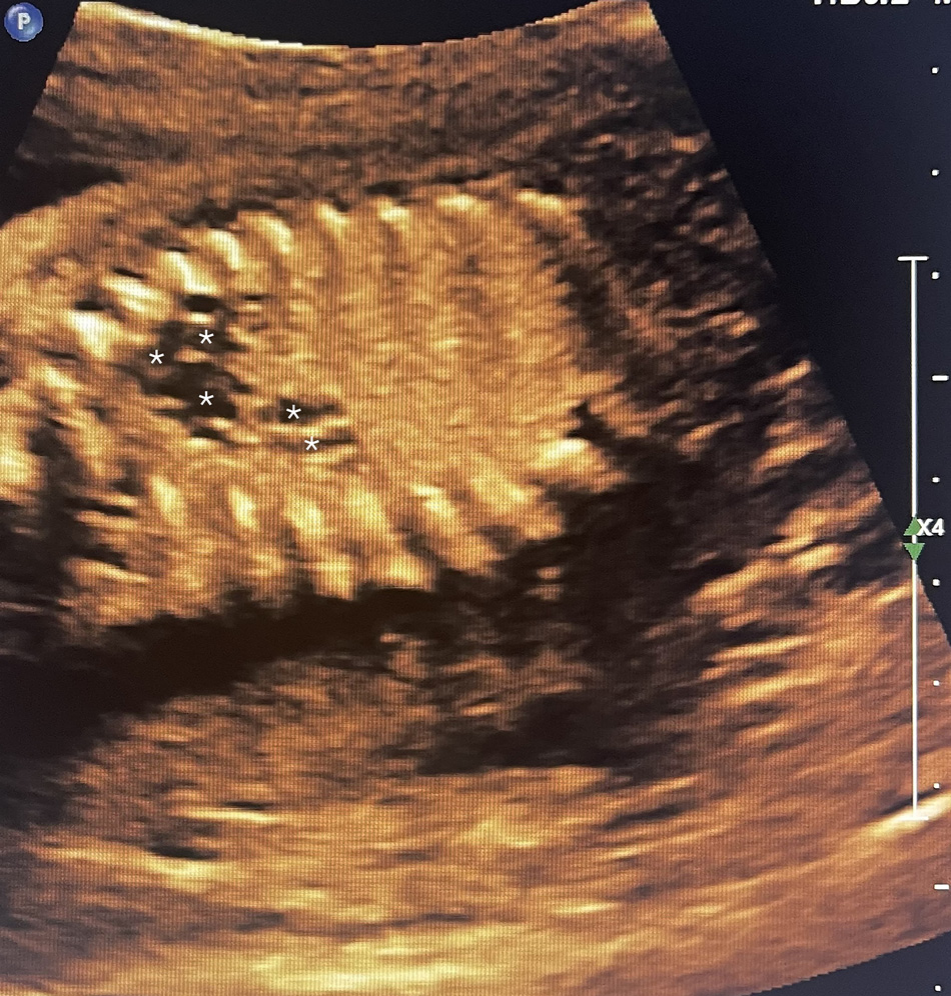
Sagittal sonographic image of the fetal heart at 26 weeks’ gestation. Note the considerably less prominent presence of cystic structures of varying sizes (mixed microcystic and macrocystic lesion) now limited to the cephalad portion of the left fetal lung (and not throughout the entire upper left lobe as depicted earlier at 21 weeks’ gestation (Fig. 1).

**Fig. 5:**
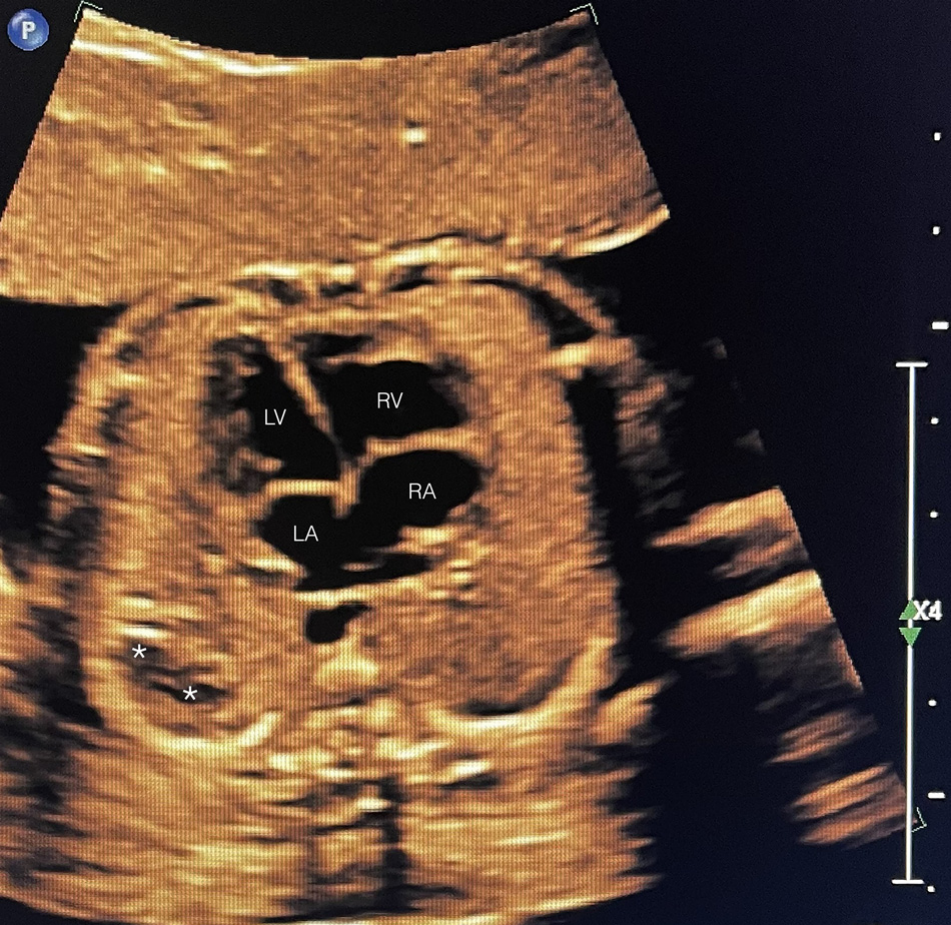
Axial sonographic image of the fetal thorax at the level of the 4-chamber heart at 26 weeks’ gestation. Note the essentially normal-positioned fetal heart representing spontaneous resolution of previously depicted marked displacement to the right, dextroposition. Note also the now normal appearing right fetal lung (previously compressed, which was barely visible at earlier 21 weeks’ gestation (compare with Fig. 2).

**Fig. 6:**
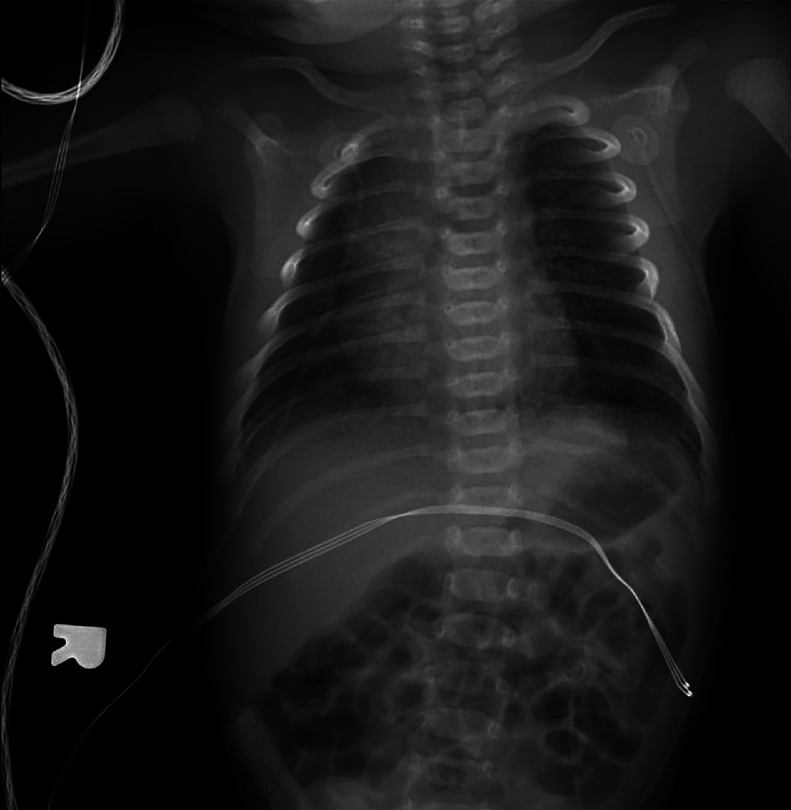
Frontal radiograph of the chest and abdomen on Day 1 of life demonstrating no evidence of cystic lung lesions or dextroposition of the fetal heart.

A 39 year-old P3 with a BMI of 29, was followed during her fourth pregnancy. Her previous pregnancies were uneventful with 3 previous spontaneous deliveries at term. She had no significant medical history. Her pregnancy was dated by an ultrasound at 11 weeks’ gestation, consistent with her last menstrual period. She was Rubella and Varicella immune. Following Genetic counseling due to advanced maternal age (AMA) she was found carrier negative for spinal muscular atrophy (SMA), and Fragile X. Cell-free DNA screening (NIPS) was negative for aneuploidy and select microdeletions. Mid-trimester maternal serum alpha-fetoprotein (MSAFP) was within normal limits at 0.91 MoM. Sonographic evaluation at 21 weeks’ gestation, revealed a singleton, appropriate for gestational age (AGA), vertex-presenting fetus. Intrathoracic anatomy disclosed multiple cystic structures of varying sizes of the left fetal lung, consistent with extensive congenital pulmonary airway malformation (CPAM, Stocker classification type 1 or 2) (Fig. 1). Detailed color Doppler imaging did not disclose a feeding vessel. The otherwise normal structured fetal heart (including normal left and right cardiac outflow tracts) was markedly displaced to the right, dextroposition (Fig. 2). The cardiomediastinal shift was considered severe, with the cardiomediastinal shift angle measuring approximately 20 degrees (Fig. 3) [3]. The fetal right lung was not visualized well. Given the marked dextroposition of the fetal heart, concern was raised regarding potential subsequent hypoplasia of the right fetal lung due to prolonged compression. Following extensive counseling the patient elected to continue her pregnancy, and declined amniocentesis. At 26 weeks’ gestation at sonographic follow up, despite the continued presence of extensive CPAM of left fetal lung, complete spontaneous regression of the dextroposition of the fetal heart and severe cardiomediastinal shift were clearly evident (Fig. 4, Fig. 5). Repeat ultrasound assessments confirmed continued gradual in-utero regression of the lung lesion. At 39 and 4/7 weeks’ gestation following spontaneous rupture of membranes, the patient spontaneously delivered a vigorous male neonate weighing 3175 grams. Apgar scores were 9 and 8 at 1 and 5 minutes, respectively and umbilical artery pH = 7.26, and base excess = - 4.3. The infant was considered to have mild transient tachypnea of the newborn, and received supplement oxygen by CPAP, from which it was weaned at 6 hours of life. Pediatric cardiology echocardiography confirmed normal cardiac structure. Chest X-ray performed on Day 1 of life demonstrated no evidence of cystic lung lesions or dextroposition of the fetal heart (Fig. 6). Given that resection of CPAM in an asymptomatic infant is controversial, definitive neonatal chest MR imaging was deferred until 2 months of age [6]. Both mother and infant were discharged in good health on postpartum Day 3.

**Fig. 7:**
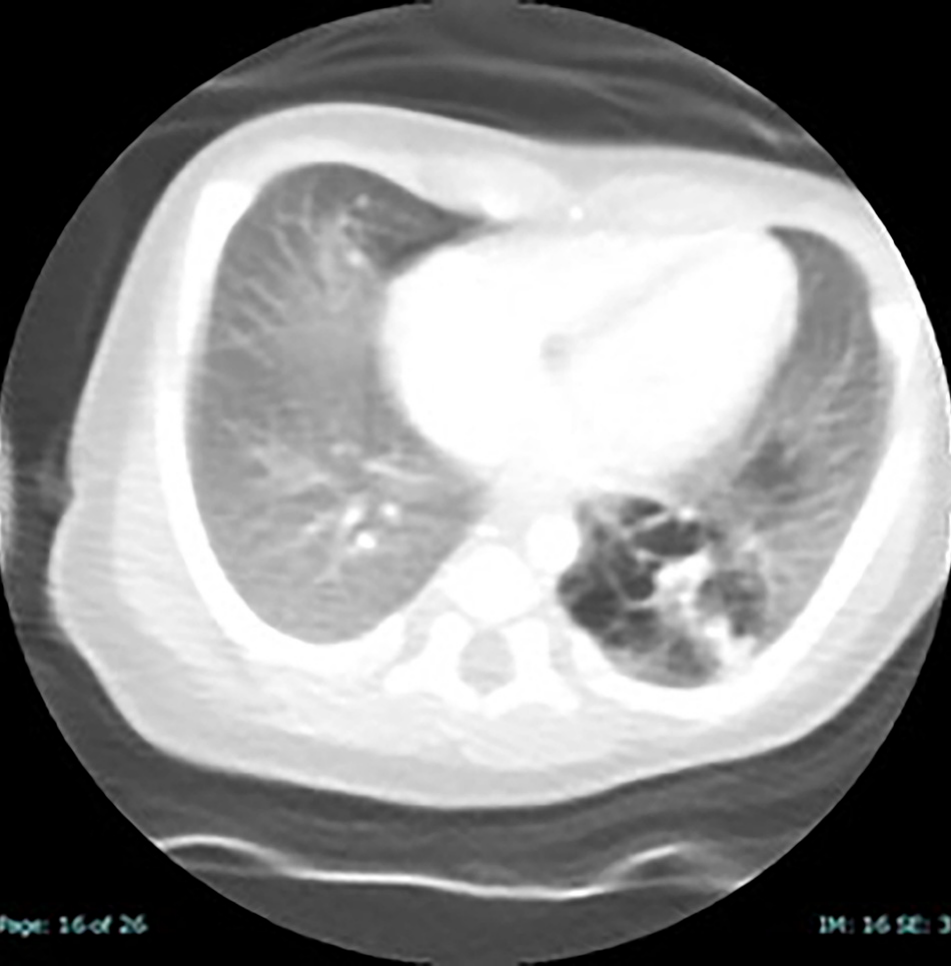
Axial CT angiogram performed on Day 47 of life demonstrating predominantly cystic air-filled CPAM hybrid lesion of the left lower lobe.

**Fig. 8:**
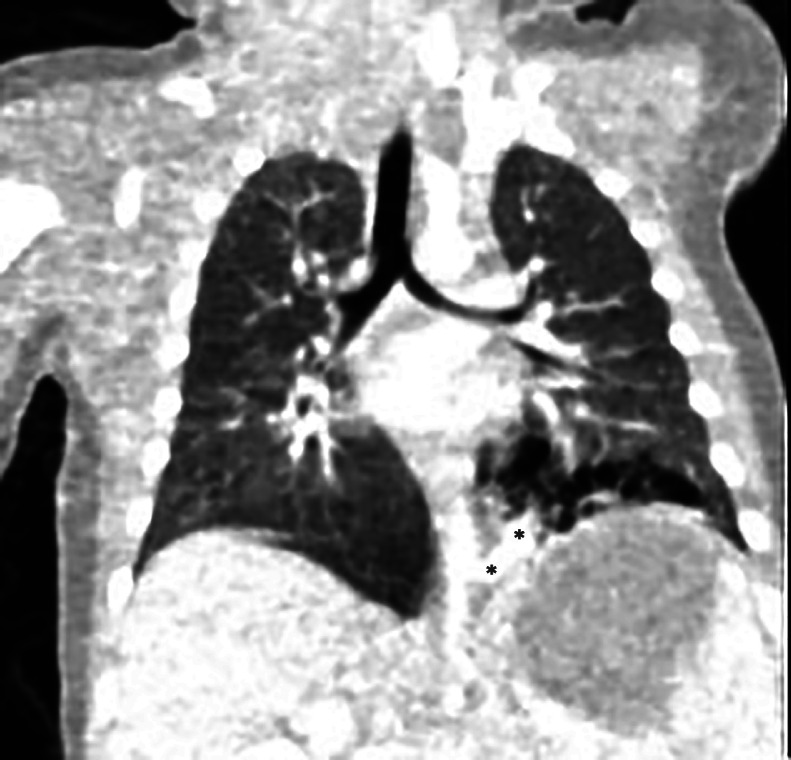
Coronal CT angiogram at the level of the aorta depicting the feeding vessel of the BPS and (marked with black asterisks) and air-filled CPAM component the hybrid lesion of the left lower lobe.

On Day 47 of life CT (with and without contrast) revealed evidence of a feeding vessel arising from the thoracic aorta indicating a hybrid lung lesion (intralobar BPS with CPAM) was documented (Fig. 7, Fig. 8).

**Fig. 9:**
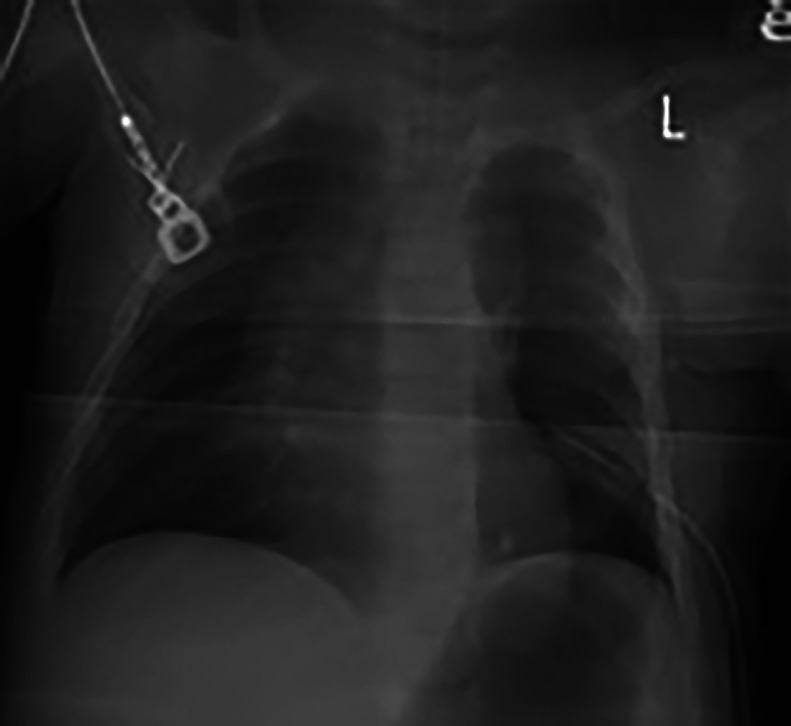
Postoperative radiograph of the neonatal chest demonstrating air-filled left lung. Note chest tube and surgical clips.

**Fig. 10:**
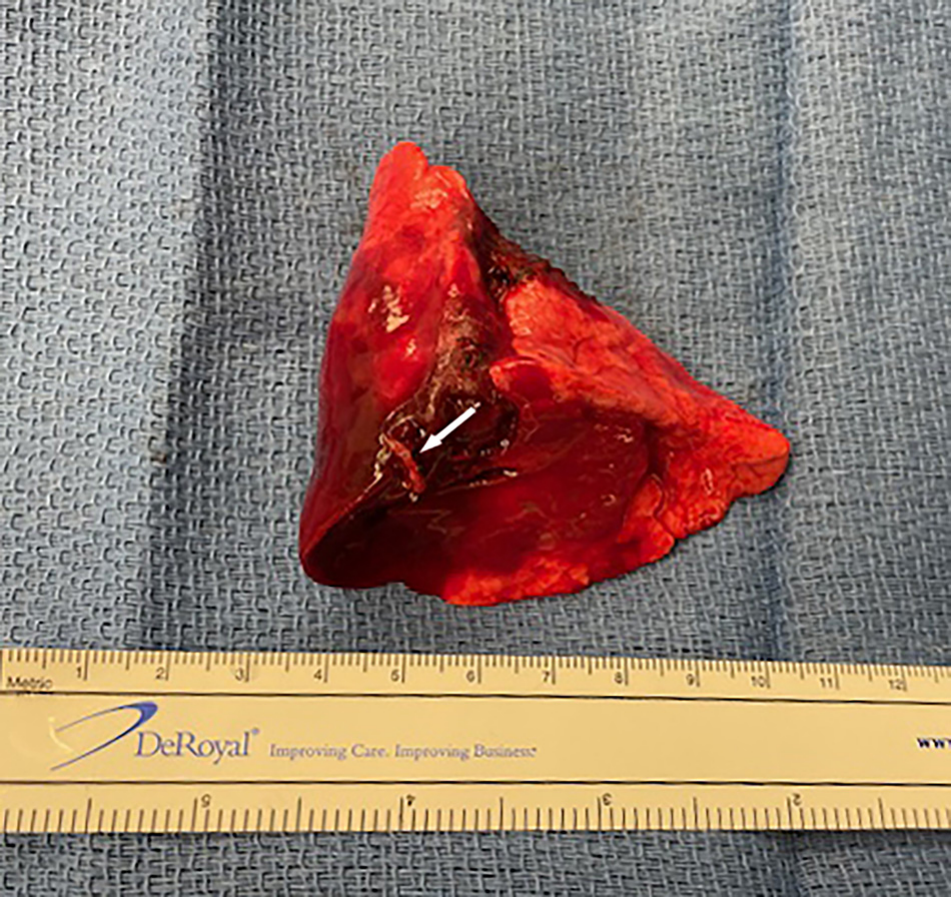
Postoperative left lower lobe surgical specimen. Note arrow pointing to feeding vessel of the BPS component of the hybrid lung lesion, which emanated from the thoracic aorta (see Fig. 8).

The parents were counseled by Pediatric Surgery regarding the possible association of BPS and subsequent infection or malignancy and accordingly on Day 58 of life, under general anesthesia with endotracheal intubation, through a left thoracotomy, uneventful resection of the left lower lobe was performed. Postoperative chest X-ray depicted an air-filled left lung (Fig. 9). Pathology examination confirmed the hybrid lesion of the lower left lobe (Fig. 10).

## Discussion

Dextrocardia infers that the main axis of the fetal heart from the base to the apex along the intraventricular septum is directed inferiorly and to the right, and may include other internal organs positioned on the opposite side [dextrocardia and situs inversus (mirror-image dextrocardia) or dextrocardia and situs solitus, with only the heart on the opposite side with other internal organs unaffected [7,8].

Dextrocardia is associated with an increased likelihood of associated structural anomalies mainly structural cardiac disease, the incidence of which varies according to the atrial situs [[7], [8], [9]].

In contrast, dextroposition of the fetal heart refers to an anatomically normal fetal heart that is displaced within the right hemithorax [10]. Etiologies may include: congenital pulmonary airway obstruction (CPAM), deformities of the chest wall, deformities of the diaphragm, right lung hypoplasia, pneumonectomy, eventration of the diaphragm, hemidiaphragmatic paralysis, diaphragmatic hernia or pneumomediastium, and scimitar syndrome (dextroposition in situs solitus due to right pulmonary hypoplasia) [11].

Hydrops in the presence of CPAM portends an increased risk for fetal or neonatal death, although the precise mechanism of the development of hydrops fetalis in association of CPAM remains unclear [3,4]. Mediastinal compression leading to increased venous pressure and decreased venous vascular return causing nonimmune hydrops has been proposed [12]. Notwithstanding, this concept has been contested, as other studies have found no association between subjective severity of cardiomediastinal shift and adverse perinatal outcome [13,14].

Shulman et al, in 2019 described a novel objective measurement of cardiomediastinal shift angle (CMSA) in cases of CPAM, and assessed the relationship of this parameter with subsequent adverse perinatal outcome and hydrops [3]. In a retrospective analysis of 85 cases referred to their institution, eighteen of the 85 cases (21.2%) experienced adverse perinatal outcomes, and hydrops [3]. While CMSA was not found to correlate with lesion laterality, lesion subtype (microcystic, macrocystic or mixed microcytic macrocystic), or gestational age, increasing CMSA was found to be associated with hydrops [3]. Adjusted analyses found each 10-degree increase in CMSA was associated with increased odds of an adverse perinatal outcome (adjusted odds ratio) [aOR] 2.2, (95% confidence interval [CI]: 1.4-3.3) and hydrops (aOR, 3.0, 95% CI: 1.5-6.1) [3].

In our case, upon initial sonographic diagnosis at 21 weeks’ gestation, extensive congenital pulmonary airway malformation (CPAM) of the left fetal lung was associated with marked dextroposition of the fetal heart (Fig. 2, Fig. 3). The right fetal lung appeared considerably compressed and the CMSA measured approximately 20 degrees (Fig. 3) indicating a concern for both potential right lung hypoplasia and possible subsequent fetal hydrops. Four weeks later, despite the continued presence of extensive CPAM of the left fetal lung (Fig. 4), complete spontaneous resolution of the previous dextroposition of the fetal heart was noted (Fig. 5). This was considered a reassuring occurrence suggesting that earlier compression of the fetal right lung had abated, and possibly may be indicative of initial, gradual (although not yet appreciable) regression of the extensive CPAM of the left fetal lung. Continued sonographic assessments at 2-week intervals confirmed continued gradual in-utero regression of the CPAM. Resolution of CPAM was considered (with no cystic lung lesions noted) at neonatal chest imaging following delivery (Fig. 6).

Notwithstanding, following neonatal CT angiogram and depiction of a feeding vessel emanating from the thoracic aorta indicating a hybrid lesion (intralobar BPD and CPAM) of the lower left lobe, resection of the left lower lobe of the neonatal lung was performed due to concerns of possible subsequent infection or malignancy [15,16]. Although CPAM and BPS represent distinctly separate entitles, hybrid lung lesions consisting of CPAM and BPS have been reported previously [[17], [18], [19]].

Systematic review of the English literature (PubMed, Google Scholar, and Medline, 1966-2025) using the search terms “prenatal ultrasound”, “fetal echocardiography”, “dextroposition”, “dextrocardia”, “congenital pulmonary airway malformation”, “congenital cystadenomatoid malformation”, “bronchopulmonary sequestration”, “hybrid lesion”, and “cardiomediastinal shift angle”, confirms that, prenatal sonographic findings of prominent dextroposition of the fetal heart (including resolution of a CMSA measurement) preceding decrease in size of a hybrid fetal lung lesion, have not been reported previously.

Our case demonstrates that spontaneous resolution of dextroposition of the fetal heart may precede later decrease in the extent of a large hybrid fetal lung lesion, and hence may possibly be considered a reassuring prognostic sign despite the presence of continued presence of this lesion.

## Patient consent

Please be note that we have obtained our patient's informed consent for publishing our case report entitled: Mid-trimester resolution of marked dextroposition of the fetal heart preceding regression of extensive left fetal lung lesion consisting of hybrid congenital pulmonary airway malformation (CPAM) and bronchopulmonary sequestration (BPS).
